# Discovery of Novel Potential Insecticide-Resistance Mutations in *Spodoptera frugiperda*

**DOI:** 10.3390/insects15030186

**Published:** 2024-03-11

**Authors:** Yuhao Cai, Huilin Chen, Mengfan Hu, Xuegui Wang, Lei Zhang

**Affiliations:** 1 Department of Entomology, China Agricultural University, Beijing 100193, China; caucaiyu@163.com (Y.C.); hmf1130@163.com (M.H.); 2 State Key Laboratory of Crop Gene Exploration and Utilization in Southwest China, Sichuan Agricultural University, Chengdu 611130, China; chenhuilin5256@163.com; 3 College of Agriculture, Sichuan Agricultural University, Chengdu 611130, China

**Keywords:** resistance monitoring, amplicon sequencing, molecular docking, pest management, novel mutations

## Abstract

**Simple Summary:**

The fall armyworm (FAW) is a pest that can cause severe damage to crops, particularly maize and cotton, resulting in agricultural losses. It has developed resistance to various types of insecticides due to certain gene mutations in its body. Our study aims to investigate how these genes affect the pest’s sensitivity to insecticides. We utilized amplicon sequencing to analyze 21 sites within six related genes and identified both known and unknown mutations. Subsequently, molecular docking simulations were employed to assess the impact of these mutations on the binding ability between insecticides and proteins. Our findings indicate that these mutations reduce the binding ability, potentially contributing to increased insecticide resistance. Thus, our study could unveil the variation and evolution of resistance genes in FAW.

**Abstract:**

The fall armyworm (FAW), *Spodoptera frugiperda*, is a worldwide agricultural pest that invaded China in 2018, and has developed resistance to multiple insecticides. The evolution of insecticide resistance is facilitated by mutations of target genes responsible for conferring resistance. In this study, amplicon sequencing analyzed 21 sites in six resistance genes. In addition to known mutations, unknown variants were also found, including novel variants: F290C (*ace-1* gene, 0.1% frequency), I1040T/V (*CHSA* gene, 0.1% frequency), A309T (*GluCl* gene, 0.1% frequency), and I4790T/V (*RyR* gene, 0.1% frequency). Additionally, molecular docking was employed to investigate the impact of the aforementioned new mutations on insecticide binding to proteins. The analyses indicated that the binding abilities were reduced, similar to the resistance mutations that were reported, implying these novel mutations may confer transitional resistance. This study may provide a foundation for understanding the functions of these novel mutations in the evolutionary processes that drive the emergence of insecticide resistance in this invasive species.

## 1. Introduction

The fall armyworm (FAW), *Spodoptera frugiperda*, is a widespread pest that can severely damage crops, especially maize and cotton, resulting in significant agricultural losses. The use of chemical insecticides is a common approach to control the population of this pest, but resistance to these chemicals poses a major problem throughout diverse regions [1,2,3,4,5].

Several identified mutations on different genes that contribute to resistance to various insecticides have been uncovered. These insecticides include organophosphate, carbamate, and diamide. Research findings suggest that the mutations A201S and F290V in the acetylcholinesterase gene (*ace-1*) of FAW and other insect species are associated with increased insecticide resistance to organophosphate insecticides [6,7,8,9,10]. Pyrethroid insecticides targeting the voltage-gated sodium channel gene (*VGSC*) bind to and disrupt their normal functioning, resulting in paralysis and death in insects. Mutations including L1014F, L932F, and T929I in *VGSG* contribute to pyrethroid resistance [11,12]. The glutamate-gated chloride channel gene (*GluCl*) mutations A309V and G314D make arthropods resistant to commonly used insecticides such as ivermectin, avermectin, and neonicotinoids [13]. Moreover, the G326E mutation within *GluCl* in *Tetranychus urticae* significantly increases resistance to the insecticide avermectin [14]. The nicotinic acetylcholine receptor (nAChR) is a complex protein composed of multiple subunits. Specific point mutations within nAChR subunits are linked to insect resistance to neonicotinoid insecticides, verified in insects such as *Nilaparvata lugens*, *Tuta absoluta*, and several aphids. Mutation (G275E) of the nAChR α6 subunit is associated with high levels of resistance to spinosyns in *T. absoluta* [15], while a three-amino-acid deletion in the transmembrane domain of the nAChR α6 subunit confers high-level resistance to spinosad in *Plutella xylostella* [16]. The Y151S mutation in *N. lugens* results in hindered binding ability to imidacloprid and resistance to various neonicotinoid insecticides [17,18]. In the case of *Myzus persicae* and *Aphis gossypii*, the R81T mutation in the β1 subunit weakens the effect of imidacloprid, causing resistance [19,20,21,22]. In addition, the L80S mutation may also be associated with imidacloprid resistance in *A. gossypii* [23]. The I1040M mutation identified in the chitin synthase A gene (*CHSA*) of FAW provided resistance to lufenuron, a CHS inhibitor impacting chitin biosynthesis [24]. A similar study discovered that the I1042M mutation in the *CHS1* gene of *P. xylostella* was associated with its resistance to numerous benzoylurea (BPU) insecticides, including diflubenzuron, flufenoxuron, lufenuron, and flucycloxuron [25]. This mutation is in the same position as the I1017F mutation, which is known to grant resistance to etoxazole in *T. urticae* [26]. The I1017F mutation in *Frankliniella occidentalis* was also associated with resistance to lufenuron [27]. Single- or multiple-point mutations in the insect ryanodine receptor gene (*RyR*) can render various classes of insecticides ineffective [28,29,30]. For example, in *P. xylostella*, the I4790M and G4946E mutations of the *RyR* gene can result in resistance to diamide insecticides [31,32,33]. Studies have demonstrated that resistance to diamides in *T. absoluta* and *Chilo suppressalis* is conferred by *RyR* target-site mutations, resulting in amino acid substitutions at the two primary sites of G4946 and I4790 [34,35].

Unlike previous studies, amplicon sequencing has allowed for the detection of genetic variations that occur at low frequencies and are linked to insecticide resistance. This study employed sensitive amplicon sequencing to extensively analyze target genes within FAW populations across different regions of the Sichuan Province, China. Notably, several novel mutations were discovered. Moreover, molecular docking analysis determined that these novel mutations potentially contribute to insecticide resistance by reducing the ability of insecticides to bind target proteins, similar to mechanisms conferred by previously identified resistance variants, which can be considered transitional resistance mutations (from sensitive to resistant). This study provides a foundation for understanding the development of insecticide resistance mechanisms in the fall armyworm, shedding light on the potential evolutionary processes that drive the emergence of resistance in this invasive species.

## 2. Materials and Methods

### 2.1. Source of Insects

The FAW populations utilized in this experiment were obtained in 2022 from various locations in Sichuan Province, China, including Cangxi County, Dechang County, Huidong County, Miyi County, Nanbu County, and Renhe Town. Samples were collected and provided by the College of Agronomy at Sichuan Agricultural University [36]. The larvae were fed specially prepared food, which contains agar strips, cornmeal, soybean flour, yeast powder, propionic acid, vitamin C, B-complex vitamins, sorbic acid, citric acid, roxithromycin tablets, and sugar. After hatching, the larvae were transferred to small containers containing food. Once they reached the third-instar stage, each larva was transferred to an individual small plastic tube with artificial food, to limit cannibalism. The adults were given a 10% honey solution as their food source. To ensure that eggs and pupae remained uncontaminated, they were treated with a solution of 0.2–0.3% sodium hypochlorite. All developmental stages were carefully examined under controlled conditions, with a temperature of 26 ± 1 °C, relative humidity ranging between 70% and 80%, and a light–dark cycle of 16 h of light and 8 h of darkness. Second-instar larvae were specifically selected and preserved in 95% ethanol for future gDNA extraction.

### 2.2. Amplicon Library Preparation and Sequencing

**Figure 1:**
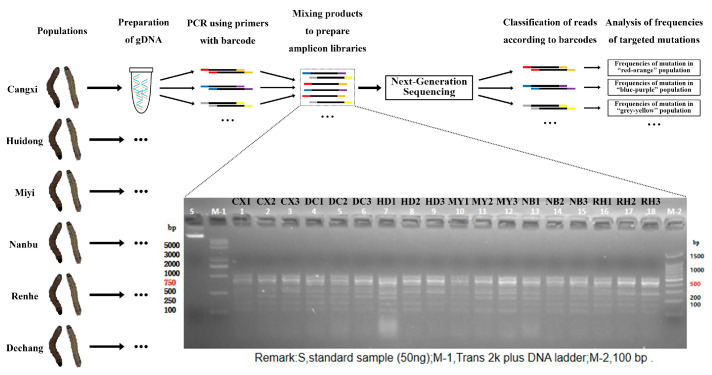
Amplicon sequencing workflow: library preparation, sequencing, and agarose gel electrophoresis results. The serial numbers 1–18 in the sub figure correspond to the following sample names: CX1, CX2, CX3, DC1, DC2, DC3, HD1, HD2, HD3, MY1, MY2, MY3, NB1, NB2, NB3, RH1, RH2, and RH3. These samples represent different FAW amplicon libraries built from Cangxi (CX1-CX3), Dechang (DC1-DC3) Huidong (HD1-HD3), Miyi (MY1-MY3), Nanbu (NB1-NB3) and Renhe (RH1-RH3). Under ultraviolet irradiation, clear DNA bands were observed for all the 18 samples. The distribution of bands was uniform, and their intensity did not decrease, indicating that the electrophoresis test results of the 18 libraries containing amplicons were satisfactory and met the quality standards required for amplicon sequencing.

Genomic DNA was obtained from 30 s-instar larvae from across six populations of FAW employing the CTAB reagent, with three repetitions. Each gDNA template which represented a population was prepared from the 30 larvae mentioned above. To detect reported target site mutations associated with insecticide resistance, specific primers were constructed based on previously identified sites, and tagged primers were included for PCR amplification of each sample (Table S1). The amplification of target fragments was conducted using the 2× Rapid Taq Master Mix reagent with the following conditions: the 50 μL PCR reaction contained 25 μL of 2× Rapid Taq Master Mix, 2 μL of each of 10 μM forward and reverse primers, and 0.5 μg of gDNA template, and the remainder was made up of ddH2O. The PCR products were quantified, and 2.5 μg of amplified products from 10 target sites from each sample group were mixed in centrifuge tubes to generate 18 mixed amplicons (Table S2). These mixture were examined using agarose gel electrophoresis (Figure S5). The mixture for sequencing was sent to the Novogene company (Beijing) for PCR-free library preparation and purification and Illumina Novaseq sequencing with PE250 reads, and the resulting data quality was examined (sub figure in Figure 1 for amplicon length, Table S3 for amplicon sequencing data output quality). A brief flow chart of amplicon library preparation and sequencing is shown in Figure 1.

### 2.3. Mutation-Frequency Analysis Based on Amplicon Sequencing Data

Several computational tools were employed for data processing and analysis in this study. FastQC was utilized to evaluate raw sequencing-data quality according to metrics including base error rate, quality score distribution, adapter contamination, and base length distribution to guide the subsequent preprocessing steps. Seqtk_demultiplex (parameter “-l 6”) was employed to divide sequences into different files based on barcodes and remove the 5’ end marker sequences. Cutadapt was utilized to remove 3′ end marker sequences, retain high-quality sequences lacking tags, and filter merged paired-end sequencing results. FLASH and PANDAseq programs were used to merge and splice paired-end reads through the identification of overlapping regions to enhance coverage and accuracy. The BWA MEM algorithm was utilized to align filtered data to the FAW genome reference using SAMtools to extract aligned reads in FASTA format. The FASTX-Toolkit performed deduplication and enumeration of read frequencies. To filter PCR-introduced genetic mutations, a self-written Python program was used for filtering, variation frequency analysis, and frequency calculations, retaining reads with a count ≥15 (different from the typical frequency > 1/2*n* in Sanger sequencing, where “*n*” is the sample size) to ensure sufficient high-quality reads while filtering out potential false positives. Clustal Omega performed multiple sequence alignments to detect mutations, insertions, and deletions (see Figure S1–S4).

### 2.4. Molecular Docking Analysis and Visualization

All parameters were set to their default values, except for the following modifications:

The amino acid sequences of FAW (GenBank accession numbers: XP_035429701.2 for AChE1, XP_035452745.1 for GluCl, XP_050552783.1 for CHSA, and XP_050555787.1 for RyR) were examined via protein homology modeling and tertiary structure prediction using SWISS-Model, MODELLER and Phyre2 [37,38,39]. RCSB accession numbers of templates: 5X61 (Chain A) for AChE1, Q8IPN4 (Chain A) for CHSA, 3RIA (Chain C) for GluCl, and 8UQ3 (Chain A) for RyR. SWISS-Model was used for the single subunit homology modeling of CHSA; Phyre2 was used for the single subunit homology modeling of AChE1 and RyR; and MODELLER was used for the multiple subunit homology modeling of GluCl. The 3D structures of ligand molecules acquired from PubChem were optimized using Gypsum-DL [40]. PubChem CIDs of ligands: 1982 for acephate, 6434889 for abamectin (B1a), 71777 for lufenuron, and 11271640 for chlorantraniliprole. The ADFR Suite was utilized to prepare the proteins and ligands, including the addition of polar hydrogens and Gasteiger charges. The ligands were docked into the binding pockets of the target proteins and defined surrounding mutation sites using Autodock Vina and MGLtools [41]. To predict non-covalent interactions and visualize the docking positions, PLIP, Open Babel, and Open-source PyMOL were employed [42,43].

## 3. Results

### 3.1. Resistant Mutation-Frequency Determination by Amplicon Sequencing

Based on the information presented in Table S3, the raw sequencing yield was 9.52 GB, and following filtering it was reduced to 7.99 GB, suggesting considerable data output. The changes in Raw Reads and Clean Reads were minimal, with a retention rate between 78.95 and 95.7%. There were differences in output between different samples, but the quality control indicators were proximal. The error rate was low, at 0.03–0.04%, with high Q20 and Q30 values (both higher than 86%) as well as moderate GC content of 46–48%.

#### 3.1.1. Acetylcholinesterase Gene (*ace-1*)

**Figure 2:**
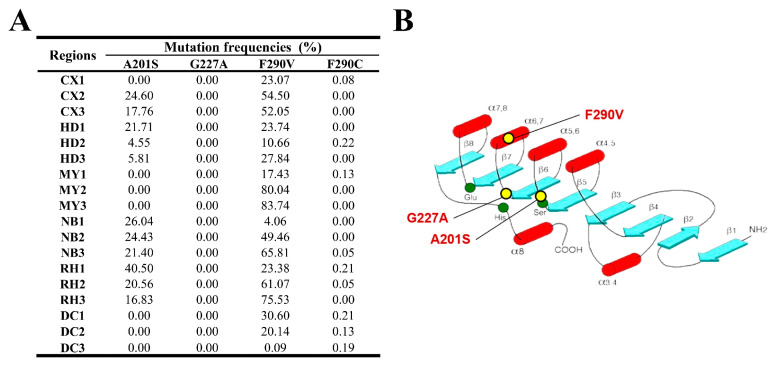
(**A**) Target-site mutation frequencies and (**B**) AChE1 architecture in FAW larvae collected from across six regions. The architecture of AChE1 is derived from Hay Dvir [44].

The results of amplicon sequencing to examine mutation frequencies in the *ace-1* gene from across six regions of Sichuan Province, China are illustrated in Figure 2A, which displays the mutation frequencies as percentages in different FAW samples collected from Cangxi (CX1–CX3), Huidong (HD1–HD3), Miyi (MY1–-MY3), Nanbu (NB1–-NB3), Renhe (RH1–-RH3), and Dechang (DC1–-DC3). The A201S mutation was identified in all regions except Miyi and Dechang, with the highest frequency of 40.5% found in Renhe and over 24.6% in Nanbu, suggesting that this mutation was primarily localized to these two regions. The G227A mutation was not identified in any region, while the F290V mutation was detected in all regions, with frequencies of over 80% in Miyi 2 and 3, and over 20% in Cangxi 1, Huidong 1, and Dechang 1. This mutation was moderately prevalent, at levels between 10 and 50% in most other samples, indicating that it was commonly distributed across the study area but primarily in Miyi, Nanbu, and Renhe.

#### 3.1.2. Voltage-Gated Sodium Channel Gene (*VGSC*)

**Figure 3:**
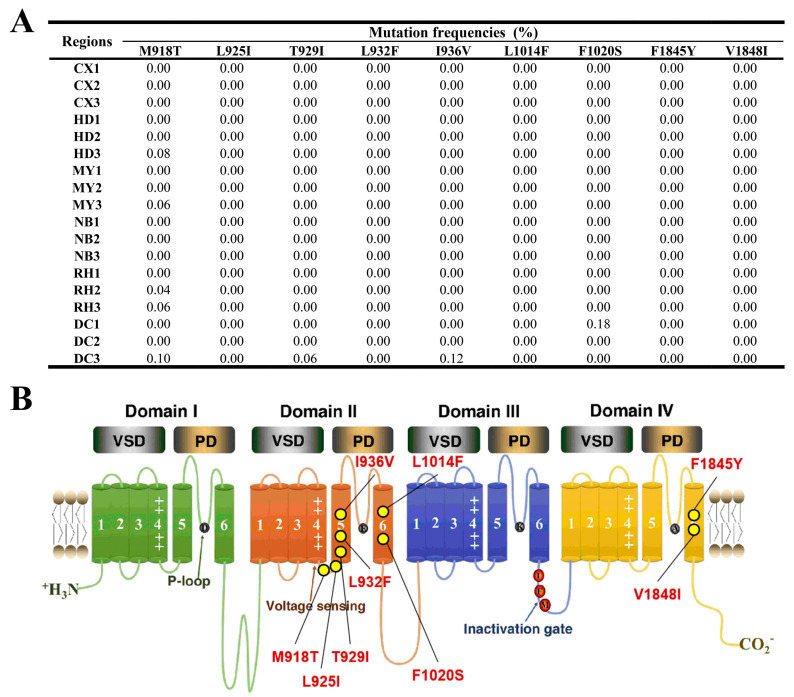
(**A**) Target-site mutation frequencies and (**B**) VGSC architecture in FAW larvae collected from across six regions. The architecture of VGSC is derived from Xing-Liang Wang and Lei Xu [45,46].

As illustrated in Figure 3A, the results of monitoring mutation frequencies in the *VGSC* gene can be observed. After examining the figure, it was determined that sample DC1 exhibited the highest F1020S mutation frequency, of 0.18%. The mutations M918T, T929I, I936V, and F1020S were identified in only a few regions, including HD3, MY3, RH2, RH3, and DC3, with frequencies ranging from 0.04% to 0.12%. The remaining nine mutation sites exhibited a frequency of 0% across all the monitored regions. In DC3, the mutation frequencies were 0.10% for M918T, 0.06% for T929I, and 0.12% for I936V. HD3 exhibited a M918T mutation frequency of 0.08%, while MY3 had a frequency of 0.06% for M918T, RH2 had a frequency of 0.04% for M918T, and RH3 had a frequency of 0.06% for M918T.

#### 3.1.3. Glutamate-Gated Chloride Channel Gene (*GluCl*)

**Figure 4:**
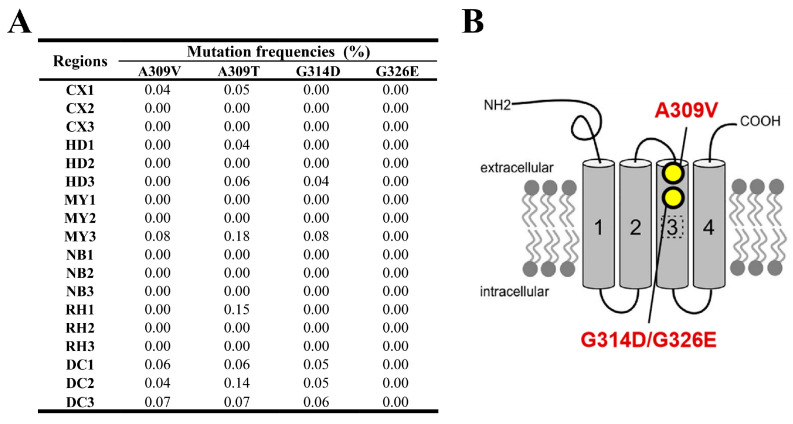
(**A**) Target-site mutation frequencies and (**B**) GluCl architecture in FAW larvae collected from across six regions. The architecture of GluCl is derived from Richard H. French-Constant and Wenxin Xue [47,48].

The findings from monitoring the mutation frequencies in the GluCl gene are presented in Figure 4A. This figure depicts how the A309V mutation frequency remained below 0.07% across all regions except MY3, where it reached 0.076%. The G314D mutation was identified at low frequencies in regions such as HD3, MY3, and DC1-3, ranging from 0.04% to 0.08%. With the exception of A309T, the frequencies of other *GluCl* resistance target-site mutations (A309V, G314D, and G326E) were comparatively low, predominantly below or at 0.1%, and the G326E mutation was not identified at any location.

#### 3.1.4. Nicotinic Acetylcholine Receptor Gene (*nAChR*)

**Figure 5:**
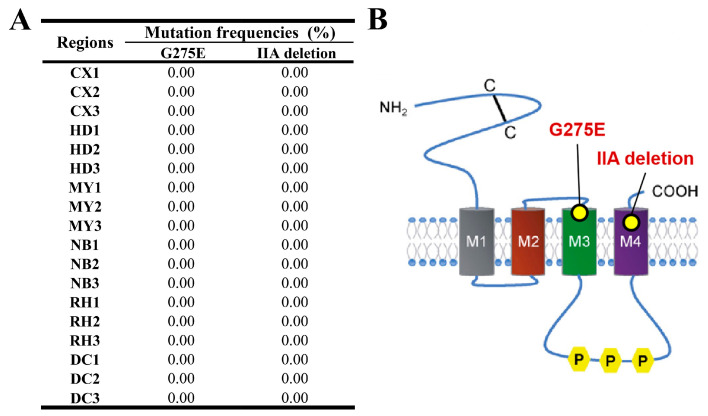
(**A**) Target-site mutation or deletion frequencies and (**B**) nAChR architecture of FAW larvae collected from across six regions. The architecture of nAChR is derived from Wellington M. Silva, Julien Dupuis, and Jing Wang [15,16,49].

As depicted in Figure 5A, the monitoring of mutation or deletion frequencies in the *nAChR* gene can be observed. This figure illustrates the frequencies of the G275E resistance mutation and IIA subunit deletion within populations of FAW. As shown in Figure 5A, the frequency of the G275E mutation and IIA subunit deletion was determined to be 0% across all monitored areas and samples, indicating that there are no relevant mutations or deletions present within these regions. The investigation performed across the six regions did not identify the presence of the G275E resistance mutation or the deletion of the IIA subunit in FAW.

#### 3.1.5. Chitin Synthase A Gene (*CHSA*)

**Figure 6:**
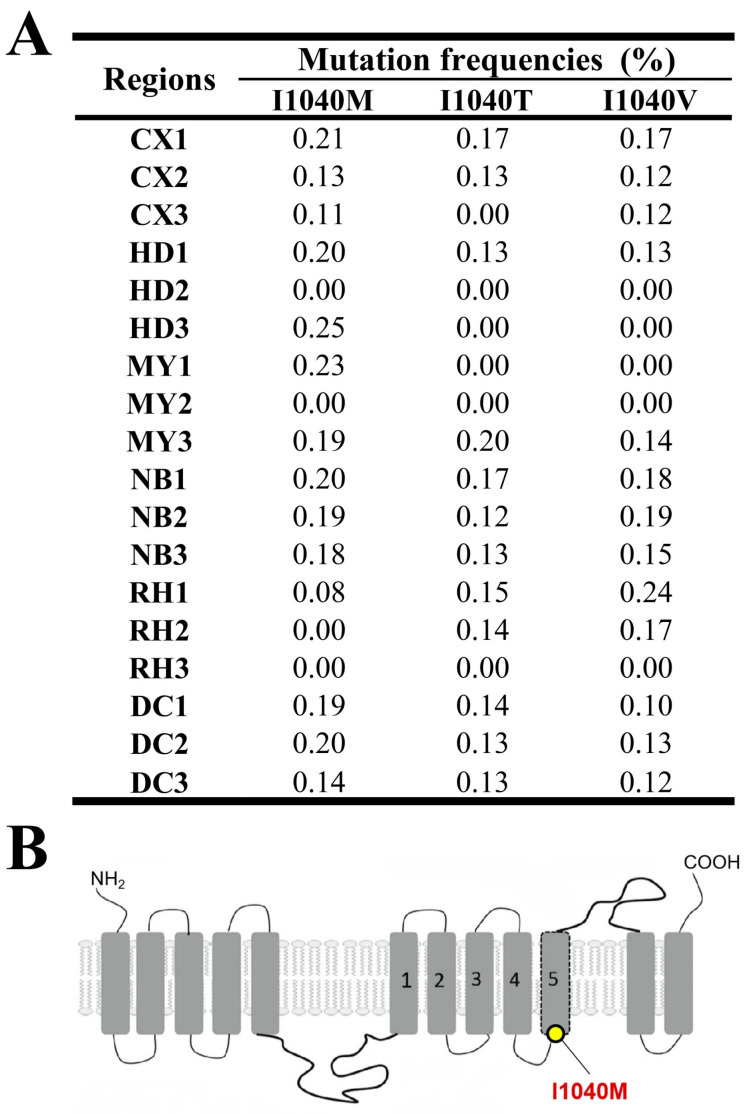
(**A**) Target-site mutation frequencies and (**B**) CHSA architecture of FAW larvae collected from across six regions. The architecture of CHSA is derived from Sheng-Lan Lv and T. Van Leeuwen [24,50].

The results of monitoring mutation frequencies in the CHSA gene are shown in Figure 6A. The frequencies of these mutations ranged from 0% to 0.25% across all monitoring areas. Specifically, in Cangxi, the frequency of the I1040M mutation spanned from 0.11% to 0.21%, while the frequencies for I1040T and I1040V ranged between 0% and 0.17%. In Huidong, the frequencies for I1040M were between 0% and 0.25%, while both I1040T and I1040V had frequencies between 0% and 0.13%. In Miyi, the frequencies of I1040M were between 0% and 0.23%, and for I1040T and I1040V, the frequencies ranged from 0% to 0.20% and 0% to 0.14%, respectively. In Nanbu, the frequencies were similar, with I1040M ranging from 0.18% to 0.20%, I1040T between 0.12% and 0.17%, and I1040V between 0.15% and 0.19%. In Renhe, the frequencies were as follows: I1040M ranged from 0% to 0.08%, I1040T between 0% and 0.15%, and I1040V between 0% and 0.24%. Finally, in Dechang, the frequencies were 0.14% to 0.20% for I1040M, 0.13% to 0.14% for I1040T, and 0.10% to 0.13% for I1040V. The I1040M mutation was frequently identified in Cangxi, Huidong, Nanbu, and Dechang, whereas I1040T and I1040V were more sporadic.

#### 3.1.6. Ryanodine Receptor Gene (*RyR*)

**Figure 7:**
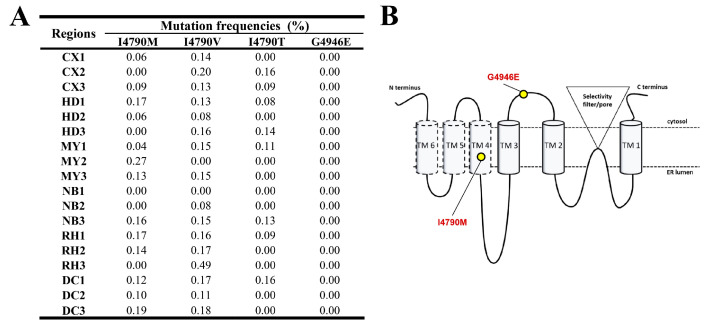
(**A**) Target-site mutation frequencies and (**B**) RyR architecture of FAW larvae collected from across six regions. The architecture of RyR is derived from Lei Guo and Bartek Troczka [31,33].

The frequencies of insecticide resistance-related mutations in the *RyR* gene of FAW populations are depicted in Figure 7A. As highlighted in the figure, the mutations I4790M, I4790V, and I4790T had relatively higher frequencies across all sites compared to G4946E, which was not detected. Specifically, in Cangxi, the occurrence of I4790M ranged from 0.00 to 0.09%, I4790T was between 0 and 0.16%, and I4790V was between 0.13 and 0.20%. In Huidong, I4790M ranged from 0 to 0.17%, I4790T from 0 to 0.14%, and I4790V from 0.08 to 0.16%. In Miyi, the occurrence of I4790M was between 0.04 and 0.27%, I4790T was between 0 and 0.11%, and I4790V was between 0 and 0.15%. Nanbu exhibited frequencies of I4790M of 0 to 0.16%, I4790T of 0 to 0.13%, and I4790V of 0 to 0.15%. Renhe exhibited frequencies of I4790M from 0 to 0.17%, I4790T from 0 to 0.09%, and I4790V from 0.16 to 0.49%. Finally, in Dechang, frequencies were 0.10 to 0.19% for I4790M, 0 to 0.16% for I4790T, and 0.11 to 0.18% for I4790V.

### 3.2. Evaluation of the Influence of Resistance-Related Gene Mutations on the Binding Affinity between Insecticide Target Proteins and Insecticides through Molecular Docking

#### 3.2.1. Acetylcholinesterase 1

**Figure 8:**
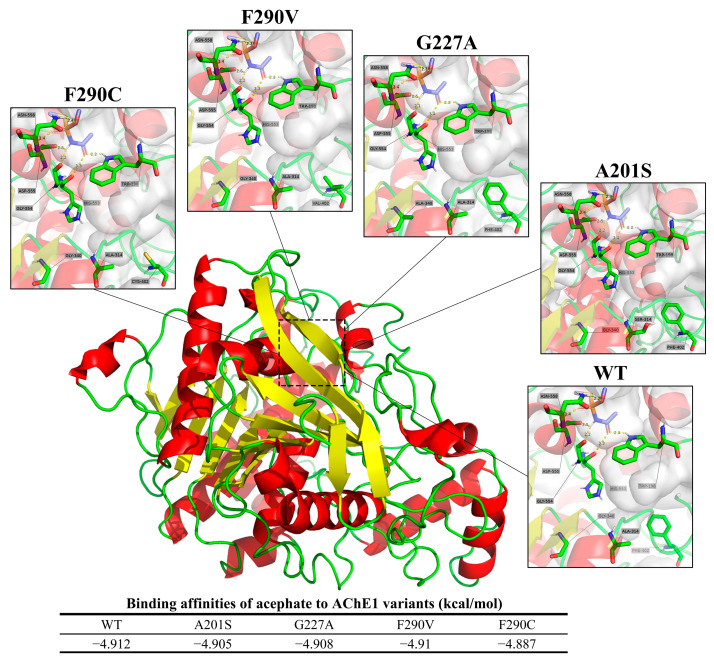
Predicted binding orientations of acephate to wild-type and mutated AChE1 from FAW.

The predicted binding modes of acephate to both the wild-type (WT) and mutated AChE1 proteins from FAW are depicted in Figure 8. In the WT protein, acephate forms hydrogen bonds with Trp198, Gly554, and Asn558 at distances of 2.3 Å, 2.2 Å, and 2.3 Å, respectively. The A201S mutation maintains similar hydrogen bonding, with 2.2 Å, 2.2 Å, and 2.3 Å bond lengths. Analogously, the G227A mutation preserves these interactions at distances of 2.2 Å, 2.1 Å, and 2.3 Å, respectively. The F290V mutation forms hydrogen bonds at lengths of 2.2 Å, 2.2 Å, and 2.3Å, respectively. However, the F290C mutation shows a slight variation in the bond lengths. Trp198, Gly554, and Asn558 form hydrogen bonds at lengths of 2.2 Å, 2.2 Å, and 2.4 Å, respectively. Generally, the binding patterns remain predominantly unchanged across the wild-type and mutant proteins, except for a slight increase in the bond length involving Asn558 in the F290C mutation.

The binding affinities between AChE1 and acephate are expressed in terms of kcal/mol. The affinity values for the different AChE1 variants are as follows: WT, −4.912 kcal/mol; A201S, −4.905 kcal/mol; G227A, −4.908 kcal/mol; F290V, −4.910 kcal/mol; and F290C, −4.887 kcal/mol.

#### 3.2.2. Glutamate-Gated Chloride Channel

**Figure 9:**
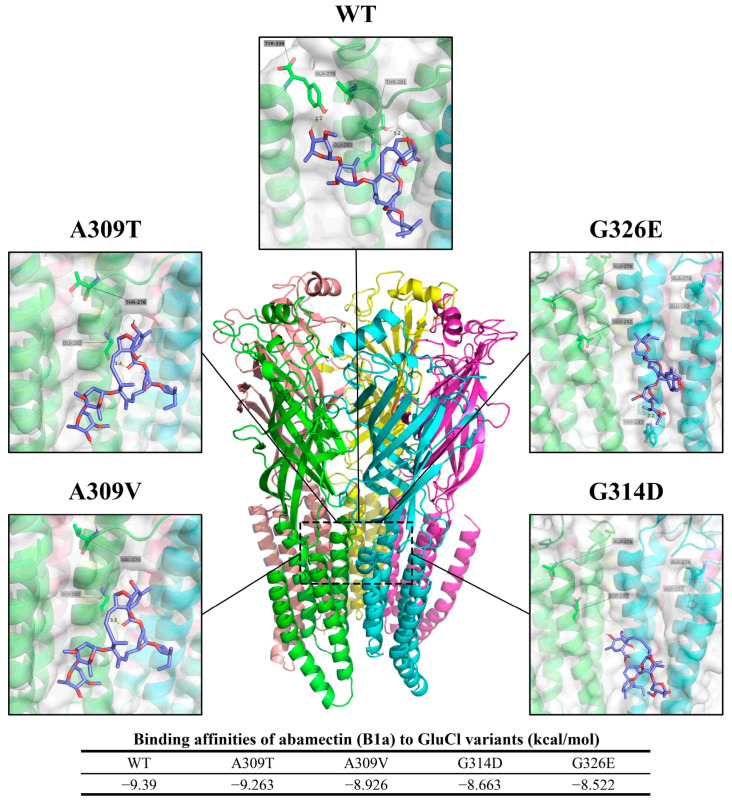
Predicted binding orientations of abamectin (B1a) to wild-type and mutated GluCl from FAW.

The predicted binding modes of abamectin (B1a) to both the wild-type (WT) and mutated GluCl proteins from FAW are depicted in Figure 9. In the WT protein, Thr 281 forms a hydrogen bond with a distance of 3.2 Å, while Tyr 339 forms a hydrogen bond with a distance of 2.2 Å. For the A309V mutation, Gly 282 forms a hydrogen bond at a distance of 3.4 Å. Correspondingly, for the A309T mutation, Gly 282 forms a hydrogen bond at a distance of 3.5 Å, and for G326E, Trp 237 forms a hydrogen bond at a distance of 2.2 Å.

The binding affinities between GluCl and abamectin (B1a) are expressed in terms of kcal/mol. The affinity values for the different CHSA variants are as follows: WT, −9.390 kcal/mol; A309T, −9.263 kcal/mol; A309V, −8.926 kcal/mol; G314D, −8.663 kcal/mol; and G326E, −8.522 kcal/mol.

#### 3.2.3. Chitin Synthase A

**Figure 10:**
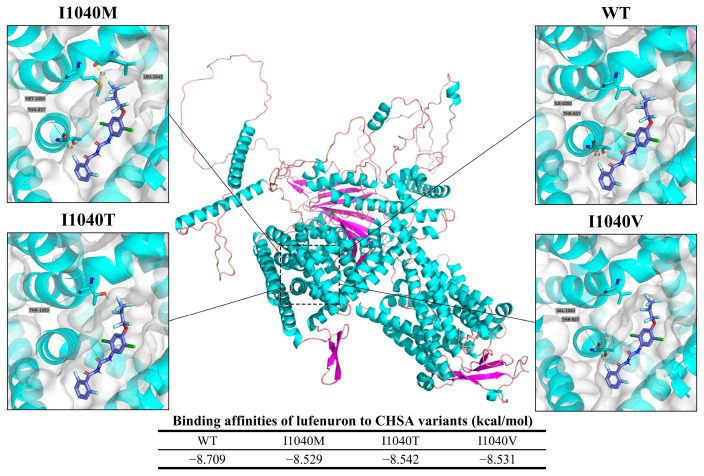
Predicted binding orientations of lufenuron to wild-type and mutated CHSA from FAW.

The predicted binding modes of lufenuron to both the wild-type (WT) and mutated CHSA proteins from FAW are depicted in Figure 10. The interaction between lufenuron and CHSA is highlighted in the figure. In the WT protein, Thr 937 forms a bond with a distance of 3.4 Å. In the case of the I1040M mutation, Thr 937 still forms a halogen bond with a distance of 3.4 Å, but Leu 1047 forms a halogen bond with a distance of 3.5 Å. Similarly, for the I1040V mutation, Thr 937 forms a halogen bond with a distance of 3.4 Å.

The binding affinities between CHSA and lufenuron were estimated through the use of AutoDock Vina and were expressed in terms of kcal/mol. The affinity values for the different CHSA variants are as follows: WT, −8.709 kcal/mol; I1040M, −8.529 kcal/mol; I1040T, −8.542 kcal/mol; and I1040V, −8.531 kcal/mol.

#### 3.2.4. Ryanodine Receptor

**Figure 11:**
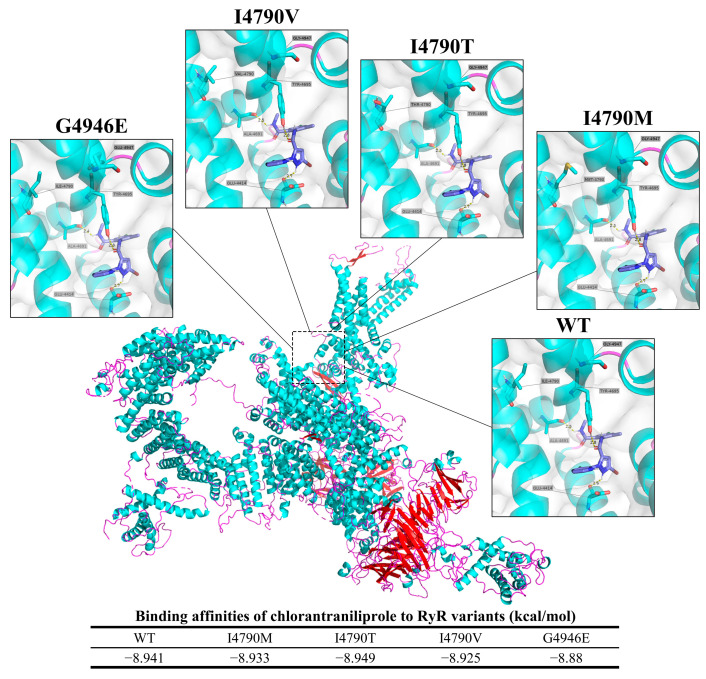
Predicted binding orientations of chlorantraniliprole to wild-type and mutated RyR from FAW.

The predicted binding modes of chlorantraniliprole to both the wild-type (WT) and mutated RyR proteins from FAW are depicted in Figure 11. In the WT protein, Ala 4691 forms a hydrogen bond with a distance of 2.5 Å, and Tyr 4695 forms a hydrogen bond with a distance of 2.8 Å, while Glu 4414 forms a halogen bond with a distance of 2.6 Å. For the I4790M/T/V mutation, Ala 4691 forms a hydrogen bond with a distance of 2.5 Å and Tyr 4695 forms a hydrogen bond with a distance of 2.8 Å, while Glu 4414 forms a halogen bond with a distance of 2.7 Å. For the G4946E mutation, Ala 4691 forms a hydrogen bond with a distance of 2.4 Å and Tyr 4695 forms a hydrogen bond with a distance of 2.8 Å, whereas Glu 4414 forms a halogen bond with a distance of 2.7 Å.

The binding affinities between RyR and chlorantraniliprole are expressed in terms of kcal/mol. The affinity values for the different RyR variants are as follows: WT, −8.941 kcal/mol; I4790M, −8.933 kcal/mol; I4790T, −8.949 kcal/mol; I4790V, −8.925 kcal/mol; and G4946E, −8.880 kcal/mol.

## 4. Discussion

The primary advantages of high-throughput sequencing are that it can substantially improve productivity, reduce the cost of monitoring, and support large-scale population or mutational studies [51]. This examination employed sensitive amplicon sequencing to generate high-quality sequencing data from 18 samples, as confirmed through quality control analyses. Sequencing reports suggested the ideal data output and high performance across extraction, library construction, and sequencing procedures. While outputs varied across samples, quality metrics revealed consistently high accuracy, as evidenced by low error rates accompanied by high Q20/Q30 scores. Over 78% of raw reads were retained following filtering, with moderately balanced GC content. These sequencing runs produced minimally altered, low-error data suitable for downstream analysis (see Table S3 for detailed information).

The FAW is a widespread, invasive pest that may develop resistance to chemical insecticides due to excessive or improper use, as well as migration from areas with higher resistance. Therefore, monitoring resistance and delaying development are required for effective long-term insecticide use [52]. This research involved an analysis of FAW populations across six regions of Sichuan Province, China, aiming to detect the frequencies of mutations in 21 sites of six closely related target genes associated with resistance through amplicon sequencing using strict filtering parameters to eliminate false positive results. This allows for the determination of a more comprehensive and accurate understanding of the resistance status and evolutionary trends of FAW in the specific area.

Based on previous studies, the A201S, G227A, and F290V mutations of acetylcholinesterase 1 (AChE1) within FAW will lead to resistance to organophosphate and carbamate insecticides [11,12,53]. A recent study in Sichuan Province’s Miyi region conducted biological assays and found high chlorpyrifos resistance in FAW [36], consistent with Wu et al.’s (2015) finding that certain mutations in AChE may confer chlorpyrifos resistance in *Apolygus lucorum* [54]. Replacement of the amino acid phenylalanine with valine was identified as reducing the available space within the acyl binding site for organophosphates, reducing binding [53]. The studies collectively indicate that mutations impacting the active binding site can diminish insecticide sensitivity. In our study, the A201 and F290 sites of the acetylcholinesterase gene (*ace-1*) exhibited high mutation frequencies in the FAW populations across the six surveyed areas. The A201S mutation was predominantly found in Nanbu and Renhe at a frequency of 20%, while F290V was widely distributed across the entire area, but was most common in Miyi County, at a frequency of 40% (Figure 2A). These findings are consistent with the results reported by Li Yan et al. on the Sichuan population in 2020 [55], even though the frequency of the A201S mutation slightly increased from 18% to 20% and the frequency of the F290V mutation decreased from 56% to 40%. The F290C mutation, with an average frequency of 0.07%, represents a novel finding as this mutation has not been reported in other investigated species nor in FAW populations (Figure 2A). Throughout molecular docking analysis, it was observed that the A201S, G227A, and F290V/C mutations displayed similar binding orientations with comparable effects on the binding affinity to acephate, suggesting that they may make similar contributions to the development of inherited resistance within FAW (Figure 8). Additionally, it was noted that the F290C mutation exhibited a more pronounced reduction in binding ability compared to the F290V mutation. This indicates that the F290C mutation is a newly identified mutation linked to resistance, which could impact the binding of acephate.

In a study of Central Colombian FAW populations, it was determined that this species is more likely to develop resistance to lambda-cyhalothrin in the field, due to its inherent strong genetic resistance [56]. Mutation of certain sites within the *VGSC* gene, such as M918T in *Aphis gossypii*, L1014F and M918T in *Myzus persicae*, T929I, F1860Y, and V1863I in *Plutella xylostella*, L932F in *Pediculus capitis*, and L1014F in *Musca domestica* has been linked to resistance to various insecticides such as neonicotinoids, DDT and pyrethroids, in these species [46,57,58,59,60,61]. A 2013 study demonstrated that mutations in the *VGSC* gene of FAW, previously identified in other arthropods, resulted in resistance to pyrethroid insecticides [11]. Based on research conducted by Wang et al., the FAW population in Sichuan Province has experienced a reduction in its resistance ratio (RR value) to lambda-cyhalothrin, from 4.23 in 2020 to 1.20 in 2021, with its RR value for indoxacarb being below 3.00 [62]. In this study, it has been revealed that the occurrence of resistance-associated mutations in the *VGSC* genes of FAW in Sichuan Province was minimal (Figure 3A), with the frequency of these mutations being below 0.1% in most areas, suggesting that the FAW population in this province may be currently susceptible to pyrethroid insecticides.

GluCl is the target of many macrocyclic insecticides, including emamectin benzoate, abamectin, and ivermectin. Studies have demonstrated that interference with the *GluCl* gene of *Tetranychus cinnabarinus*, *M. separata*, and *Bemisia tabaci* can alter their sensitivity to abamectin [63,64,65,66]. The G326E mutation of the *GluCl3* gene in *Tetranychus urticae*, as well as the A309V and G315E mutations in the *GluCl* gene in *P. xylostella*, confer resistance to abamectin, affecting these two species through mutations in their respective *GluCl* genes [67,68,69]. Previous studies examining the impact of abamectin on *P. xylostella* GluCl and ivermectin on *Caenorhabditis elegans* GluCl, suggest that the mutations G314D and G326E hinder the binding of macrocyclic insecticides through steric hindrance. In contrast, the A309V mutation impacts the allosteric binding site [67,70]. In 2020 and 2021, the FAW population’s resistance ratio to emamectin benzoate in Sichuan Province was reduced from 4.67 to 2.00, and in 2022 the RR value was below 2.44 [36,62]. In this study, as Figure 4A depicts, the frequency of resistance-site mutations, including A309V and G315E in the FAW *GluCl* gene, was very low (less than 0.1%). Therefore, the FAW population in these areas of Sichuan Province currently does not have moderate or high resistance to the aforementioned insecticides. According to molecular docking analysis, it was determined that mutations G314D and G326E in the GluCl protein exhibited a noticeable effect on its binding affinity (Figure 9). These mutations disrupted the formation of a hydrogen bond between the original protein chain (Gly 282 residue) and abamectin. Furthermore, the A309T mutation demonstrated a reduced effect on weakening binding capacity compared to the A309V mutation. This suggests that the A309T mutation may serve as an intermediary stage during the progression from sensitivity to resistance.

This investigation demonstrates that the G275E modification of the nicotine acetylcholine receptor (nAChR), alongside the absence of three specific amino acids (IIA) in the α6 subunit, can cause resistance against spinosyns [15,16]. Though prior studies have indicated that the FAW in Sichuan Province has developed resistance to spinetoram (RR, 4.32–18.05-fold) [36], which targets nAChR, no mutations in the *nAChR* gene were observed in this study, suggesting that high resistance has not developed within the population in Sichuan Province (Figure 5).

In a study on the FAW population throughout Sichuan Province, Lv et al. identified the fact that resistance to lufenuron had increased from 1.8 in 2019 to 4.0 in 2020, but they did not identify the I1040M/I1040F mutations linked to this resistance [24]. The *CHS* gene interference prevents the ecdysis and pupation of FAW [71]. This study identified three mutations at the I1040 site of the *CHSA* gene in the FAW population in Sichuan Province, but these were found at a low frequency, of around 0.1% (Figure 6). This suggests that the FAW population is likely to have developed some degree of resistance to chitin synthase inhibitor insecticides. Molecular docking analysis uncovered similar patterns in the variants of CHSA’s I1040, resulting in a decreased ability to bind to lufenuron (Figure 10). Based on the docking binding model, while the mutated residues I1040M/T/V do not directly interact with lufenuron through hydrogen bonds or halogen bonds, their mutation still impacts the binding affinity.

The RyR receptor has been the primary target of several insecticides, including neonicotinoids, synthetic pyrethroids, and organophosphates. Insect RyR resistance-associated mutations have been identified, reducing the effectiveness of diamide insecticides by hundreds of times, including G4946E and I4790M [31,33,35,72,73]. Wan et al.’s research determined that there were no I4790M and G4946E mutations in the *RyR* gene of the FAW population in Sichuan Province between 2019 and 2021 [62]. Moreover, Chen et al.’s research demonstrated that some individuals in the 2022 FAW population in Sichuan Province have developed moderate resistance to chlorantraniliprole (with RR values ranging from 2.02 to 10.39) [36]. The female adults of the FAW strain with reduced susceptibility to chlorantraniliprole exhibited increased fertility while simultaneously decreasing their life span, allowing them to better withstand the effects of the pesticide [74]. In this study, the I4790M mutation with a frequency of up to 0.27% was identified, but the G4946E mutation was not detected (Figure 7). The resistance of FAW populations in Sichuan Province to chlorantraniliprole may be associated with mutations at the I4790 site of the *RyR* gene. Molecular docking analysis indicated that the RyR I4790 and G4946 variants exhibited relatively limited affinities towards chlorantraniliprole (Figure 11). The G4946E mutation had a more pronounced decrease in binding affinity to chlorantraniliprol compared to the wild-type protein, while the I4790M/T/V mutations exhibited a similar binding affinity to the wild-type protein, suggesting a similar contribution to inherited resistance development in FAW. This may be achieved by changing the target protein structure and reducing sensitivity to biamide insecticides.

Multiple mutations have been recognized as potential intermediate stages in the evolution of resistance. These mutations can initially provide limited levels of resistance, subsequently progressing and developing into higher levels. Previous studies have examined three additional positional variations (g-396 G/A, g-498 A/G, and g-768 C/G) in the *S. frugiperda ace-1* gene, which may be associated with resistance development [10,53]. In the case of *P. xylostella*, the RyR mutation I4790K/M was linked to resistance against diamide insecticides [75,76]. Similarly, in *Culex pipiens*, CHS1 mutations I1043F/L/M were associated with high levels of resistance to diflubenzuron (DFB) [77,78]. Introducing or producing mutations conferring resistance is highly likely to result in the evolution of insecticide resistance [7,79]. This study has revealed several new mutations, including A309T in GluCl, which was previously unidentified. It was observed that this mutation reduces the ability of GluCl to bind with abamectin. Additionally, other mutations, namely F290C in AChE1, I1040T/V in CHSA, and I4790T/V in RyR, were shown to weaken binding capacity. Based on these findings, it can be inferred that these novel mutations play an important role in resistance development in FAW.

## 5. Conclusions

This study has shed light on several previously unknown mutations in the insecticide-resistant target genes of FAW, which have the potential to reduce the binding affinity of insecticides to their target sites. These findings provide valuable insights into the intermediate stages of resistance evolution, from sensitive to resistant, and have implications for predicting future resistance trends and informing proactive strategies for pest control. By identifying the genetic mutations that contribute to resistance, our study lays the groundwork for proactive resistance monitoring, enabling the management of FAW resistance through the early detection and mitigation of its development. This knowledge will be crucial in ensuring sustainable agriculture practices and protecting crops from the devastating impacts of FAW infestations.

## Data Availability

The data presented in this study are available on request from the corresponding author. For data access, please contact the corresponding author at zhanglei86@cau.edu.cn.

## Supplementary Materials

The following supporting information can be downloaded at: https://www.mdpi.com/article/10.3390/insects15030186/s1, Figure S1: Multiple sequence alignment of AChE1 Proteins from major insects; Figure S2: Multiple sequence alignment of CHSA Proteins from major insects; Figure S3: Multiple sequence alignment of GluCl Proteins from major insects; Figure S4: Multiple sequence alignment of RyR Proteins from major insects; Figure S5: Electrophoresis of PCR products for construction of amplicon library; Table S1: Primers for amplicon sequencing; Table S2: Concentration of amplicon library; Table S3: Overview of amplicon sequencing data output quality.
