# Supplementary material for: Discovery of Novel Potential Insecticide-Resistance Mutations in Spodoptera frugiperda
Source: Insects. 2024 Mar 11;15(3):186. doi: 10.3390/insects15030186 (PMC10971490; doi:10.3390/insects15030186)
Supplement: Supplementary file 1 [file insects-15-00186-s001.zip › insects-2860825-supplementary.pdf]

insects

MDPI

Article

Discovery of Novel Potential Insecticide-Resistance Mutations in *Spodoptera frugiperda*

Yuhao Cai [1], Huilin Chen [2,3], Mengfan Hu [1], Xuegui Wang [2,3,*] and Lei Zhang [1,*]

1 Department of Entomology, China Agricultural University, Beijing 100193, China; caucaiyu@163.com (Y.C.); hmf1130@163.com (M.H.)
2 State Key Laboratory of Crop Gene Exploration and Utilization in Southwest China, Sichuan Agricultural University, Chengdu 611130, China; chenhuilin5256@163.com
3 College of Agriculture, Sichuan Agricultural University, Chengdu 611130, China
* Correspondence: wangxuegui@sicau.edu.cn (X.W.); zhanglei86@cau.edu.cn (L.Z.)

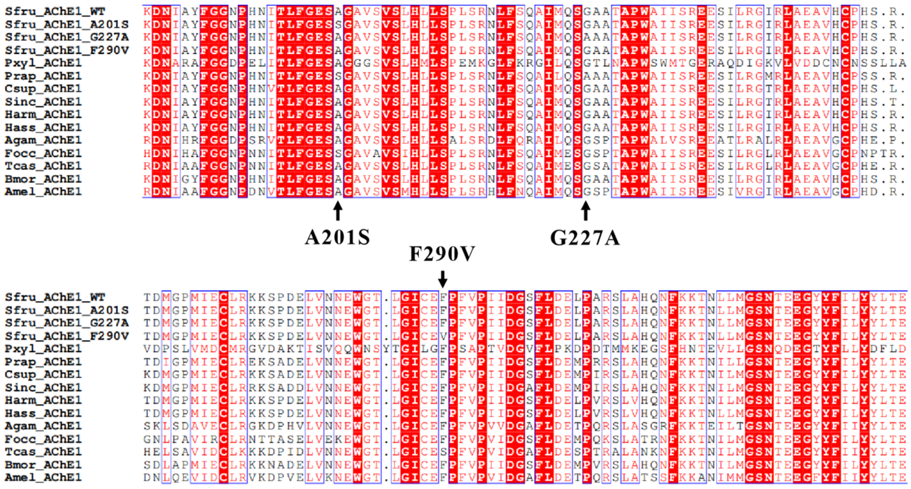

Figure S1a. Multiple sequence alignment of AChE1 Proteins from major insects. This figure shows the multiple sequence alignment of AChE1 protein sequences from different insects. Sequences were aligned using the FAW AChE1 wildtype and its three mutants (A201S, G227A, F290V) as references. GenBank accession numbers for the sequences are as follows: *Spodoptera frugiperda* (Sfru_AChE1_WT, WHS04474.1), *Plutella xylostella* (Pxyl_AChE1, AAK39639.1), *Pieris rapae* (Prap_AChE1, AQY62780.1), *Chilo suppressalis* (Csup_AChE1, ABO38111.1), *Scirpophaga incertulas* (Sinc_AChE1, AOW44163.1), *Helicoverpa armigera* (Harm_AChE1, AAY59530.1), *Helicoverpa assulta* (Hass_AChE1, AAY42136.1), *Anopheles gambiae* (Agam_AChE1, AGM16375.1), *Frankliniella occidentalis* (Focc_AChE1, KAE8737177.1), *Tribolium castaneum* (Tcas_AChE1, NP_001280548.1), *Bombyx mori* (Bmor_AChE1, ABY50088.1), *Apis mellifera* (Amel_AChE1, ANT80563.1).

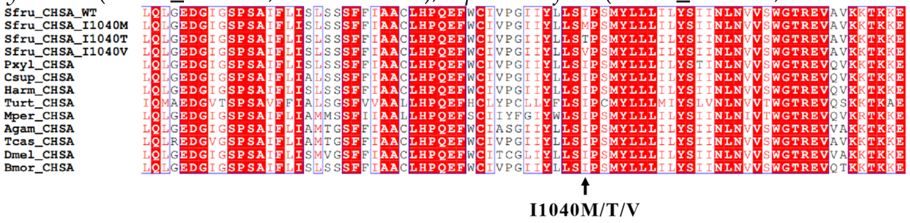

Figure S1b. Multiple sequence alignment of CHSA Proteins from major insects. This figure shows the multiple sequence alignment of CHSA protein sequences from different insects. Sequences were aligned using the FAW CHSA wildtype and its three mutants (I1040M, I1040T, I1040T) as references. GenBank accession numbers for the sequences are as follows: *Spodoptera frugiperda* (Sfru_CHSA_WT, XP_050552783.1), *Plutella xylostella* (Pxyl_CHSA, BAF47974.1), *Chilo suppressalis* (Csup_CHSA, QJF54125.1), *Helicoverpa armigera* (Harm_CHSA, AKJ54482.1), *Tetranychus urticae* (Turt_CHSA, AFG28412.1), *Myzus*

persicae (Mper_CHSA, XP_022169166.1), *Anopheles gambiae* (Agam_CHSA, XP_321336.5), *Tribolium castaneum* (Tcas_CHSA, NP_001034491.1), *Drosophila melanogaster* (Dmel_CHSA, AAG09735.1), Bombyx mori (Bmor_CHSA, XP_037877645.1).

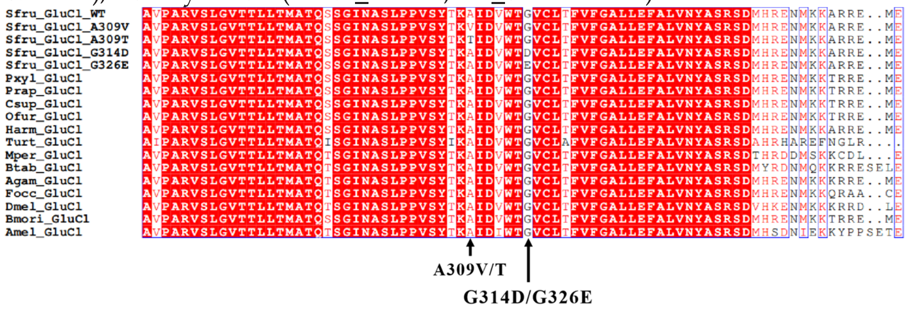

Figure S1c. Multiple sequence alignment of GluCl Proteins from major insects. This figure shows the multiple sequence alignment of GluCl protein sequences from different insects. Sequences were aligned using the FAW GluCl wildtype and its four mutants (A309V, A309T, G314D, G326E) as references. GenBank accession numbers for the sequences are as follows: *Spodoptera frugiperda* (Sfru_GluCl_WT, XP_035452745.1), *Plutella xylostella* (Pxyl_GluCl, XP_011555227.1), *Pieris rapae* (Prap_GluCl, XP_022123036.1), *Chilo suppressalis* (Csup_GluCl, UXG17918.1), *Ostrinia furnacalis* (Ofur_GluCl, XP_028160778.1), *Helicoverpa armigera* (Harm_GluCl, XP_021191008.1), *Tetranychus urticae* (Turt_GluCl, XP_015785428.1), *Myzus persicae* (Mper_GluCl, XP_022181801.1), *Bemisia tabaci* (Btab_GluCl, AVL92931.1), *Anopheles gambiae* (Agam_GluCl, AGS43089.1), *Frankliniella occidentalis* (Focc_GluCl, AZZ73574.1), *Drosophila melanogaster* (Dmel_GluCl, ABG57261.1), *Bombyx mori* (Bmori_GluCl, BAO58781.1), *Apis mellifera* (Amel_GluCl, XP_006560231.1).

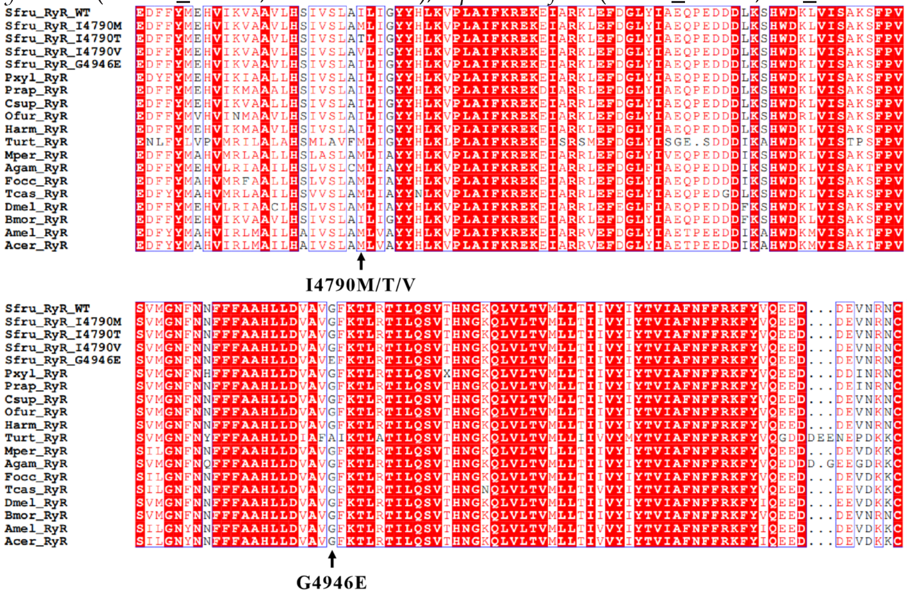

Figure S1d. Multiple sequence alignment of RyR Proteins from major insects. This figure shows the multiple sequence alignment of RyR protein sequences from different insects. Sequences were aligned using the FAW RyR wildtype and its four mutants (I4790M, I4790T, I4790T, G4946E) as references. GenBank accession numbers for the sequences are as follows: *Spodoptera frugiperda* (Sfru_RyR_WT, XP_050555787.1), *Plutella xylostella* (Pxyl_RyR, NP_001296002.1), *Pieris rapae* (Prap_RyR, XP_045485471.1), *Chilo suppressalis* (Csup_RyR, AFN70719.1), *Ostrinia furnacalis* (Ofur_RyR, AGH68757.1), *Helicoverpa armigera* (Harm_RyR, XP_049707587.1), *Tetranychus urticae* (Turt_RyR, BAK26392.1), *Myzus persicae* (Mper_RyR, XP_022160123.1), *Anopheles gambiae* (Agam_RyR, XP_318561.3), *Frankliniella occidentalis* (Focc_RyR, XP_052124088.1), *Tribolium castaneum* (Tcas_RyR, NP_001308588.1), *Drosophila melanogaster* (Dmel_RyR, NP_476991.1), *Bombyx mori* (Bmor_RyR, XP_037875957.1), *Apis mellifera* (Amel_RyR, XP_006569098.1), *Apis cerana* (Acer_RyR, XP_016920093.1).

insects MDPI

Table S1. Primers for amplicon sequencing.

Primer Name	Primer Sequence (5′ to 3′)	Mutation	Product length (bp)
Sfru-ace-1-F	ATCACGCCTGATGTACCGGGT'AATGCTG	A201S/G227A/F290V	500
Sfru-ace-1-R	CGATGTTAACCTTCCTCCGTATTGGATCC		
FAW-VGSC-F1	TGACCAGGTTCCGCAGTAGCATAGGTGA	M918T/L925I/T929I/L932F/I936V	422
FAW-VGSC-R1	CACTCAAGTTACTCCATGATCACTTTGCTTG		
FAW-VGSC-F2	ACAGTGCAGATTACGTAGACCGTTTCCCG	L1014F/F1020S	414
FAW-VGSC-R2	GCCAATCGTCGGCTACGTTACGTTTCAC		
FAW-VGSC-F3	TAGCTTATGGTATCATCAACGAGGAAGAGTG	F1845Y/V1848I	302
FAW-VGSC-R3	ACTTGAATGTCGTAATCGTCGTCTGTGAG		
FAW-GluCl-F	GATCAGGTAACGACACTCCTCACGATGG	A309V/G314D/G326E	261
FAW-GluCl-R	GTTTCGCGTGGTGTTGCTATCTGTGTCG		
FAW-nAChR-F1	CGTACGTTCACCTTGCCTCCAGATTCT	G275E	423
FAW-nAChR-R1	CAGATCAGTACAGGAACACGCATCGAAT		
FAW-nAChR-F2	AGTCAATCTACAGAGCGAACTTGTTGCA	IIA deletion(the three amino acid (IIA) deletion at the α2 subunit)	367
FAW-nAChR-R2	AGTTCCCTACGACACCATGATGTGCG		
Sfru-ChSA-F	ATGTCAATCTCCTTCGGCTATATTCTTGAT	I1040M	208
Sfru-ChSA-R	CCGTCCCTTCGTCTTCTTAACTGCCACTTC		
Sfru-RyR-F1	ACTGATACAACTCCTTCCTCTACTCTC	G4946E	151
Sfru-RyR-R1	GAGTGGTGTTTTCCGTTATGCGTGAC		
Sfru-RyR-F2	ATGAGCGACGAGGACTTCTTCTACATGGA	I4790M	102
Sfru-RyR-R2	CAAAAGCACCTTGAGATGGTAGTACCCGA		

Table Note: The label sequence for each primer is underlined.

Table S2. Concentration of amplicon library.

Sample Name	Concentration (ng/μL)	Volume (μL)	Total (μg)
CX1	36.8	94.0	3.459
CX2	44.4	105.0	4.662
CX3	50.2	100.0	5.020
DC1	50.8	78.0	3.962
DC2	45.8	93.0	4.259
DC3	49.0	89.0	4.361
HD1	80.6	70.0	5.642
HD2	55.2	87.0	4.802
HD3	53.8	83.0	4.465
MY1	33.4	96.0	3.206
MY2	48.6	81.0	3.937
MY3	57.6	65.0	3.744
NB1	60.2	85.0	5.117
NB2	36.2	90.0	3.258
NB3	46.4	65.0	3.016
RH1	48.4	75.0	3.630
RH2	49.2	80.0	3.936
RH3	51.2	85.0	4.352

Table Note: The concentration of 18 amplicon libraries varied between 33.4 and 80.6 ng/μL, and the total volume of libraries varied from 3.016 to 5.642 μg, indicating that the library reached a concentration of 24 ng/μL with a total quantity greater than 2.5 μg, meeting the necessary standards for subsequent amplicon sequencing studies.

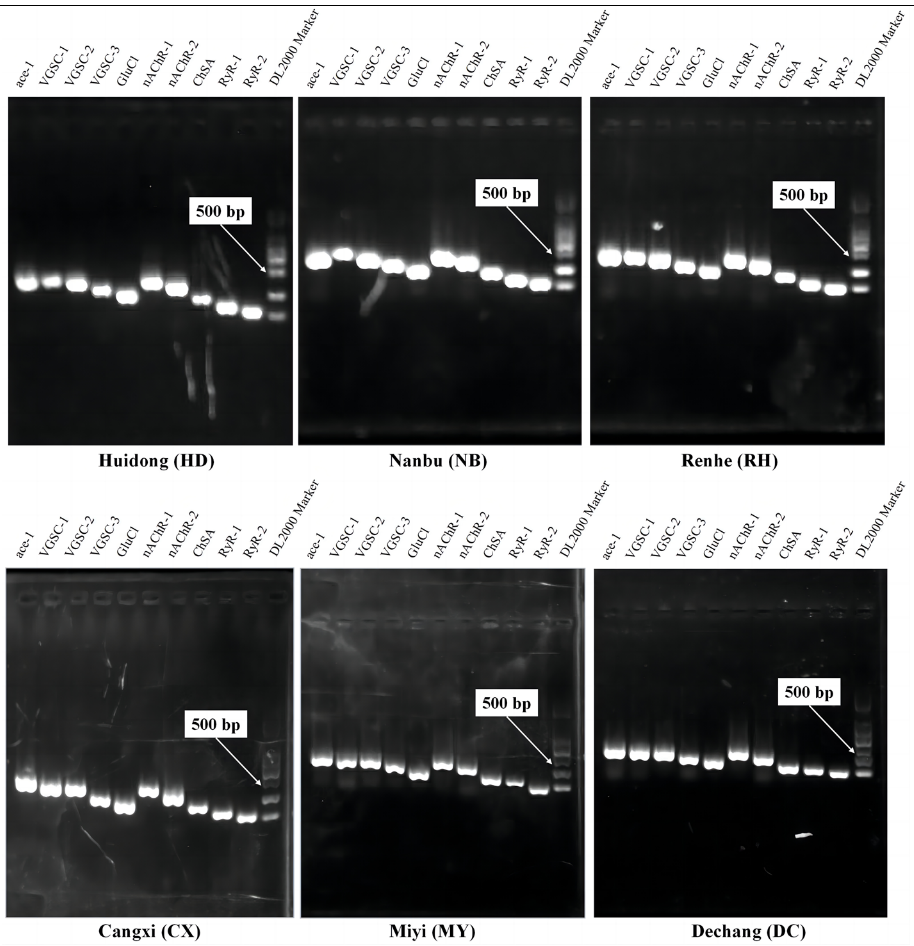

Figure S2. Electrophoresis of PCR products for construction of amplicon library. The electrophoresis bands are labeled from left to right, with each band corresponding to a specific "Primer Name" (-F pairs -R) from Table A1. Starting from the leftmost band, the mapping follows the order of rows from top to bottom in Table A1.

Table S3. Overview of amplicon sequencing data output quality.

Sample	Library Name	Raw Reads	Clean Reads	Raw Base(G)	Clean Base(G)	Effective(%)	Error(%)	Q20(%)	Q30(%)	GC(%)
CX1	FDDP230114884-1a	2575124.00	2214346.00	0.64	0.55	85.99	0.03	95.71	89.78	47.71
CX2	FDDP230114885-1a	1977974.00	1661776.00	0.49	0.42	84.01	0.03	96.01	90.19	46.55
CX3	FDDP230114886-1a	1989294.00	1756752.00	0.50	0.44	88.31	0.03	95.65	89.01	47.31
DC1	FDDP230114887-1a	2551422.00	2023382.00	0.64	0.51	79.30	0.03	95.45	89.43	47.53
DC2	FDDP230114888-1a	2480310.00	2067156.00	0.62	0.52	83.34	0.03	95.93	90.15	47.25
DC3	FDDP230114889-1a	2221740.00	1942380.00	0.56	0.49	87.43	0.03	96.02	90.22	47.02
HD1	FDDP230114890-1a	2108266.00	1675658.00	0.53	0.42	79.48	0.04	93.52	86.69	47.06
HD2	FDDP230114891-1a	1407424.00	1111192.00	0.35	0.28	78.95	0.04	93.19	86.40	47.69
HD3	FDDP230114892-1a	1902920.00	1736554.00	0.48	0.43	91.26	0.03	95.86	89.90	47.93
MY1	FDDP230114893-1a	1872842.00	1477370.00	0.47	0.37	78.88	0.04	92.65	85.72	47.78
MY2	FDDP230114894-1a	1524890.00	613476.00	0.38	0.15	40.23	0.03	93.78	87.31	46.37
MY3	FDDP230114895-1a	2136224.00	1942356.00	0.53	0.49	90.92	0.03	95.81	89.84	47.02
NB1	FDDP230114896-1a	1345378.00	1168196.00	0.34	0.29	86.83	0.04	93.78	87.13	46.31
NB2	FDDP230114897-1a	2008494.00	1721566.00	0.50	0.43	85.71	0.03	95.38	89.31	46.72
NB3	FDDP230114898-1a	2665674.00	2321344.00	0.67	0.58	87.08	0.03	95.79	89.88	46.78
RH1	FDDP230114899-1a	2476146.00	1997832.00	0.62	0.50	80.68	0.03	95.33	89.23	47.35
RH2	FDDP230114900-1a	2644544.00	2449238.00	0.66	0.61	92.61	0.03	95.86	89.96	46.66
RH3	FDDP230114901-1a	2171578.00	2078182.00	0.54	0.52	95.70	0.03	95.90	89.97	47.08
